# (Acetyl­acetonato-κ^2^ *O*,*O*′)dichlorido­bis(methano­lato-κ*O*)niobium(V)

**DOI:** 10.1107/S1600536812042638

**Published:** 2012-10-20

**Authors:** Leandra Herbst, Hendrik G. Visser, Andreas Roodt, Carla Pretorius

**Affiliations:** a Department of Chemistry, University of the Free State, PO Box 339, Bloemfontein, 9300, South Africa

## Abstract

In the title compound, [Nb(CH_3_O)_2_(C_5_H_7_O_2_)Cl_2_], a slightly distorted octa­hedral coordination geometry is observed around the Nb^V^ atom with Nb—O distances in the range of 1.8254 (16)–2.0892 (16) Å and Nb—Cl distances of 2.3997 (14) and 2.4023 (12) Å. The O—Nb—O angles vary between 81.36 (7) and 172.65 (7) °, while the *trans* Cl—Nb—Cl angle is 167.34 (2)°. There are no hydrogen bonds observed.

## Related literature

For synthetic background, see: Herbst *et al.* (2010 ▶; 2011 ▶); Davies *et al.* (1999 ▶). For applications of acetyl­acetone-type ligands in industry, see: Steyn *et al.* (1992 ▶, 1997 ▶, 2008 ▶); Otto *et al.* (1998 ▶); Roodt & Steyn (2000 ▶); Brink *et al.* (2010 ▶); Viljoen *et al.* (2008 ▶, 2009**a* ▶,b* ▶, 2010 ▶). For related niobium complexes, see: Sokolov *et al.* (1999 ▶, 2005 ▶); Anti­nolo *et al.* (2000 ▶); Dahan *et al.* (1976 ▶).
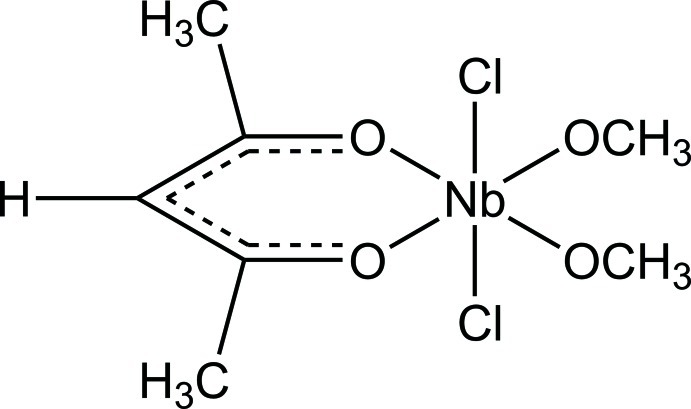


## Experimental

### 

#### Crystal data

[Nb(CH_3_O)_2_(C_5_H_7_O_2_)Cl_2_]
*M* *_r_* = 324.98
Monoclinic, 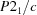

*a* = 7.7985 (2) Å
*b* = 11.6028 (3) Å
*c* = 14.6819 (2) Å
β = 111.279 (1)°
*V* = 1237.91 (5) Å^3^
*Z* = 4
Mo *K*α radiation
μ = 1.39 mm^−1^
*T* = 100 K
0.38 × 0.13 × 0.08 mm

#### Data collection

Bruker APEXII CCD diffractometer
Absorption correction: multi-scan (*SADABS*; Bruker, 2004 ▶) *T*_min_ = 0.810, *T*_max_ = 0.895
25521 measured reflections
2995 independent reflections
2873 reflections with *I* > 2σ(*I*)
*R*_int_ = 0.024

#### Refinement

*R*[*F* ^2^ > 2σ(*F* ^2^)] = 0.026
*wR*(*F* ^2^) = 0.060
*S* = 1.04
2995 reflections
131 parameters
H-atom parameters constrained
Δρ_max_ = 1.90 e Å^−3^
Δρ_min_ = −1.14 e Å^−3^

### 

Data collection: *APEX2* (Bruker, 2005 ▶); cell refinement: *SAINT-Plus* (Bruker, 2004 ▶); data reduction: *SAINT-Plus*; program(s) used to solve structure: *SIR92* (Sheldrick, 2008 ▶); program(s) used to refine structure: *SHELXL97* (Sheldrick, 2008 ▶); molecular graphics: *DIAMOND* (Brandenburg & Putz, 2004 ▶); software used to prepare material for publication: *WinGX* (Farrugia, 1999 ▶).

## Supplementary Material

Crystal structure: contains datablock(s) global, I. DOI: 10.1107/S1600536812042638/bt6846sup1.cif

Structure factors: contains datablock(s) I. DOI: 10.1107/S1600536812042638/bt6846Isup2.hkl

Additional supplementary materials:  crystallographic information; 3D view; checkCIF report

## Figures and Tables

**Fig. 1.**
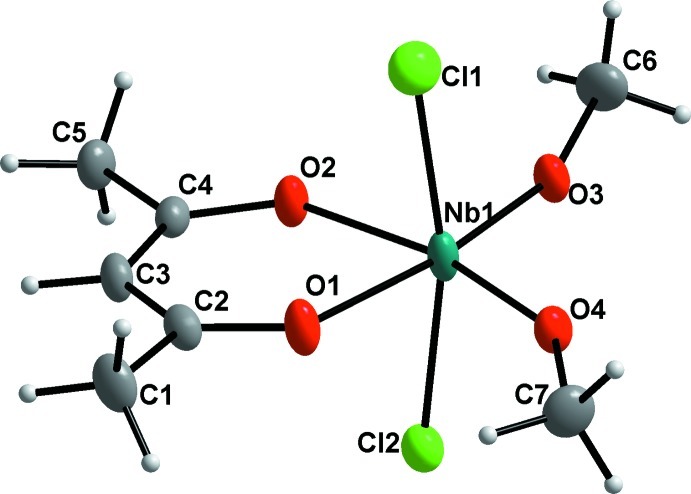
Molecular structure of the title compound. Displacement ellipsoids are drawn at the 50% probability displacement level.

## supplementary crystallographic information

### Comment

Acetylacetone and other β-diketones are strong chelating agents that find applications in homogenous catalysis and the separations industry (Steyn *et al*., 1992; 1997; Otto *et al.*, 1998; Roodt & Steyn, 2000; Brink *et al.*, 2010). This study forms part of ongoing research to investigate the interaction of transition metals used in the nuclear industry, specifically zirconium, hafnium, niobium and tantalum, with *O*,*O*'- and *N*,*O*-bidentate ligands. (Steyn *et al.*, 2008; Viljoen *et al.*, 2008; 2009**a*,b*; 2010; Herbst *et al.*, 2010; 2011).

The title complex crystallizes in the monoclinic space group *P*2_1_/*c* with *Z* = 4. The assymetric unit consists of a niobium(V) atom surrounded by two methanolate groups, two chlorido ligands and an *O*,*O*'-bonded acetylacetonato ligand (Figure 1). The octahedral environment around the niobium metal centre is slightly disordered with Nb—O distances varying between 1.8254 (16) and 2.0892 (16) Å, while the Nb—Cl distances are 2.3997 (14) and 2.4023 (12) Å respectively. The O—Nb—O angles vary between and 81.36 (7) and 172.65 (7) °, while the *trans* Cl—Nb—Cl angle is 167.34 (2) °. All the bond distances and angles are similar to other relevant niobium(V) structures (Herbst *et al.*, 2010; 2011; Sokolov *et al.*, 1999; 2005; Antinolo *et al.*, 2000 and Dahan *et al.*, 1976).

### Experimental

NbCl_5_ (0.3134 g; 1.16 mmol) was carefully dissolved in absolute methanol (5 ml) (Care: exothermic reaction). Acetylacetone (0.119 ml; 1.16 mmol) was added to the solution. The colourless solution was stirred for 1 h at room temperature and the solution was left to stand at 252 K for 24 h after which pale-yellow crystals, suitable for X-ray diffraction were obtained.

### Refinement

The methyl and aromatic H atoms were placed in geometrically idealized positions and constrained to ride on their parent atoms, with C—H = 0.95 and 0.98 Å and *U*_iso_(H) = 1.5*U*_eq_(C) and 1.2*U*_eq_(C), respectively. The highest peak is located 0.74 Å from Nb1 and the deepest hole is situated 0.65 Å from Nb1.

### Crystal data

**Table:**

[Nb(CH_3_O)_2_(C_5_H_7_O_2_)Cl_2_]	*F*(000) = 648
*M**_r_* = 324.98	*D*_x_ = 1.744 Mg m^−^^3^
Monoclinic, *P*2_1_/*c*	Mo *K*α radiation, λ = 0.71069 Å
Hall symbol: -P 2ybc	Cell parameters from 9867 reflections
*a* = 7.7985 (2) Å	θ = 2.3–32.9°
*b* = 11.6028 (3) Å	µ = 1.39 mm^−^^1^
*c* = 14.6819 (2) Å	*T* = 100 K
β = 111.279 (1)°	Cubiod, yellow
*V* = 1237.91 (5) Å^3^	0.38 × 0.13 × 0.08 mm
*Z* = 4	

### Data collection

**Table:**

Bruker APEXII CCD diffractometer	2873 reflections with *I* > 2σ(*I*)
Graphite monochromator	*R*_int_ = 0.024
φ and ω scans	θ_max_ = 28°, θ_min_ = 2.3°
Absorption correction: multi-scan (*SADABS*; Bruker, 2004)	*h* = −10→9
*T*_min_ = 0.810, *T*_max_ = 0.895	*k* = −14→15
25521 measured reflections	*l* = −19→19
2995 independent reflections	

### Refinement

**Table:**

Refinement on *F*^2^	Primary atom site location: structure-invariant direct methods
Least-squares matrix: full	Secondary atom site location: difference Fourier map
*R*[*F*^2^ > 2σ(*F*^2^)] = 0.026	Hydrogen site location: inferred from neighbouring sites
*wR*(*F*^2^) = 0.060	H-atom parameters constrained
*S* = 1.04	*w* = 1/[σ^2^(*F*_o_^2^) + (0.0177*P*)^2^ + 2.6018*P*] where *P* = (*F*_o_^2^ + 2*F*_c_^2^)/3
2995 reflections	(Δ/σ)_max_ = 0.001
131 parameters	Δρ_max_ = 1.90 e Å^−^^3^
0 restraints	Δρ_min_ = −1.14 e Å^−^^3^

### Special details

**Table:**

Experimental. The intensity data was collected on a Bruker X8 ApexII 4 K Kappa CCD diffractometer using an exposure time of 60 s/frame. A total of 1033 frames were collected with a frame width of 0.5° covering up to θ = 28.32° with 99.8% completeness accomplished.
Geometry. All s.u.'s (except the s.u. in the dihedral angle between two l.s. planes) are estimated using the full covariance matrix. The cell s.u.'s are taken into account individually in the estimation of s.u.'s in distances, angles and torsion angles; correlations between s.u.'s in cell parameters are only used when they are defined by crystal symmetry. An approximate (isotropic) treatment of cell s.u.'s is used for estimating s.u.'s involving l.s. planes.
Refinement. Refinement of *F*^2^ against ALL reflections. The weighted *R*-factor *wR* and goodness of fit *S* are based on *F*^2^, conventional *R*-factors *R* are based on *F*, with *F* set to zero for negative *F*^2^. The threshold expression of *F*^2^ > 2σ(*F*^2^) is used only for calculating *R*-factors(gt) *etc*. and is not relevant to the choice of reflections for refinement. *R*-factors based on *F*^2^ are statistically about twice as large as those based on *F*, and *R*- factors based on ALL data will be even larger.

### Fractional atomic coordinates and isotropic or equivalent isotropic displacement parameters (Å^2^)

**Table:**

	*x*	*y*	*z*	*U*_iso_*/*U*_eq_	
C1	0.5973 (4)	0.0556 (2)	0.41589 (19)	0.0291 (5)	
H1A	0.5041	0.0091	0.3698	0.044*	
H1B	0.5461	0.0949	0.4577	0.044*	
H1C	0.697	0.0073	0.4547	0.044*	
C2	0.6671 (3)	0.14217 (19)	0.36226 (16)	0.0209 (4)	
C3	0.6842 (3)	0.25776 (19)	0.38971 (15)	0.0217 (4)	
H3	0.6428	0.2801	0.439	0.026*	
C4	0.7590 (3)	0.34165 (18)	0.34795 (15)	0.0189 (4)	
C5	0.7749 (3)	0.4644 (2)	0.38144 (17)	0.0256 (5)	
H5A	0.8962	0.4925	0.3915	0.038*	
H5B	0.7531	0.4689	0.4416	0.038*	
H5C	0.6856	0.5104	0.3326	0.038*	
C6	1.0638 (4)	0.3334 (2)	0.1093 (2)	0.0351 (6)	
H6A	1.0417	0.4104	0.1263	0.053*	
H6B	1.0441	0.3302	0.0409	0.053*	
H6C	1.1884	0.3119	0.1467	0.053*	
C7	0.7155 (4)	−0.0737 (2)	0.12959 (19)	0.0315 (5)	
H7A	0.6627	−0.0836	0.1788	0.047*	
H7B	0.8087	−0.131	0.1379	0.047*	
H7C	0.6213	−0.0817	0.0661	0.047*	
O1	0.7114 (2)	0.10264 (14)	0.29243 (12)	0.0260 (3)	
O2	0.8199 (2)	0.32042 (13)	0.27942 (12)	0.0236 (3)	
O3	0.9420 (2)	0.25678 (14)	0.12973 (12)	0.0249 (3)	
O4	0.7944 (2)	0.03654 (13)	0.13835 (12)	0.0235 (3)	
Cl1	1.11377 (9)	0.13058 (6)	0.32569 (5)	0.03473 (14)	
Cl2	0.51757 (8)	0.23365 (5)	0.10144 (4)	0.02774 (12)	
Nb1	0.82643 (3)	0.178209 (17)	0.198700 (15)	0.02255 (7)	

### Atomic displacement parameters (Å^2^)

**Table:**

	*U*^11^	*U*^22^	*U*^33^	*U*^12^	*U*^13^	*U*^23^
C1	0.0387 (13)	0.0244 (11)	0.0336 (12)	0.0020 (10)	0.0241 (11)	0.0074 (9)
C2	0.0234 (10)	0.0220 (10)	0.0208 (10)	0.0033 (8)	0.0122 (8)	0.0047 (8)
C3	0.0271 (11)	0.0233 (10)	0.0194 (10)	0.0032 (8)	0.0142 (9)	0.0006 (8)
C4	0.0204 (10)	0.0202 (10)	0.0166 (9)	0.0022 (8)	0.0074 (8)	−0.0015 (7)
C5	0.0325 (12)	0.0212 (10)	0.0277 (11)	−0.0021 (9)	0.0162 (10)	−0.0065 (9)
C6	0.0348 (13)	0.0404 (14)	0.0344 (13)	−0.0160 (11)	0.0177 (11)	−0.0021 (11)
C7	0.0391 (14)	0.0204 (11)	0.0358 (13)	−0.0076 (10)	0.0145 (11)	−0.0050 (10)
O1	0.0399 (9)	0.0187 (7)	0.0296 (8)	−0.0034 (7)	0.0247 (8)	−0.0019 (6)
O2	0.0349 (9)	0.0186 (7)	0.0246 (8)	−0.0050 (6)	0.0194 (7)	−0.0042 (6)
O3	0.0307 (8)	0.0245 (8)	0.0274 (8)	−0.0071 (7)	0.0199 (7)	−0.0046 (6)
O4	0.0291 (8)	0.0184 (7)	0.0279 (8)	−0.0037 (6)	0.0161 (7)	−0.0059 (6)
Cl1	0.0379 (3)	0.0295 (3)	0.0374 (3)	−0.0022 (2)	0.0144 (3)	−0.0032 (2)
Cl2	0.0297 (3)	0.0293 (3)	0.0283 (3)	−0.0040 (2)	0.0154 (2)	−0.0029 (2)
Nb1	0.03316 (12)	0.01790 (10)	0.02524 (11)	−0.00586 (8)	0.02096 (9)	−0.00534 (7)

### Geometric parameters (Å, º)

**Table:**

C1—C2	1.495 (3)	C6—H6A	0.96
C1—H1A	0.96	C6—H6B	0.96
C1—H1B	0.96	C6—H6C	0.96
C1—H1C	0.96	C7—O4	1.405 (3)
C2—O1	1.280 (3)	C7—H7A	0.96
C2—C3	1.393 (3)	C7—H7B	0.96
C3—C4	1.387 (3)	C7—H7C	0.96
C3—H3	0.93	O1—Nb1	2.0892 (16)
C4—O2	1.283 (3)	O2—Nb1	2.0429 (16)
C4—C5	1.497 (3)	O3—Nb1	1.8254 (16)
C5—H5A	0.96	O4—Nb1	1.8410 (17)
C5—H5B	0.96	Cl1—Nb1	2.4023 (12)
C5—H5C	0.96	Cl2—Nb1	2.3997 (14)
C6—O3	1.411 (3)		
			
C2—C1—H1A	109.5	H6B—C6—H6C	109.5
C2—C1—H1B	109.5	O4—C7—H7A	109.5
H1A—C1—H1B	109.5	O4—C7—H7B	109.5
C2—C1—H1C	109.5	H7A—C7—H7B	109.5
H1A—C1—H1C	109.5	O4—C7—H7C	109.5
H1B—C1—H1C	109.5	H7A—C7—H7C	109.5
O1—C2—C3	123.49 (19)	H7B—C7—H7C	109.5
O1—C2—C1	115.9 (2)	C2—O1—Nb1	133.05 (15)
C3—C2—C1	120.6 (2)	C4—O2—Nb1	134.78 (14)
C4—C3—C2	124.00 (19)	C6—O3—Nb1	159.91 (16)
C4—C3—H3	118	C7—O4—Nb1	146.66 (15)
C2—C3—H3	118	O3—Nb1—O4	100.77 (7)
O2—C4—C3	123.2 (2)	O3—Nb1—O2	92.28 (7)
O2—C4—C5	115.49 (19)	O4—Nb1—O2	166.62 (7)
C3—C4—C5	121.35 (19)	O3—Nb1—O1	172.65 (7)
C4—C5—H5A	109.5	O4—Nb1—O1	85.79 (7)
C4—C5—H5B	109.5	O2—Nb1—O1	81.36 (7)
H5A—C5—H5B	109.5	O3—Nb1—Cl2	97.31 (7)
C4—C5—H5C	109.5	O4—Nb1—Cl2	91.23 (6)
H5A—C5—H5C	109.5	O2—Nb1—Cl2	84.13 (5)
H5B—C5—H5C	109.5	O1—Nb1—Cl2	85.76 (6)
O3—C6—H6A	109.5	O3—Nb1—Cl1	92.06 (7)
O3—C6—H6B	109.5	O4—Nb1—Cl1	95.37 (6)
H6A—C6—H6B	109.5	O2—Nb1—Cl1	87.03 (5)
O3—C6—H6C	109.5	O1—Nb1—Cl1	83.97 (7)
H6A—C6—H6C	109.5	Cl2—Nb1—Cl1	167.34 (2)
